# Retroperitoneal lymph node dissection for testicular cancer in a patient with a double inferior vena cava

**DOI:** 10.1002/iju5.12247

**Published:** 2021-01-31

**Authors:** Akane Yamaguchi, Hiromitsu Negoro, Kosuke Kojo, Atsushi Ikeda, Tomokazu Kimura, Shuya Kandori, Akio Hoshi, Takahiro Kojima, Koji Kawai, Hiroyuki Nishiyama

**Affiliations:** ^1^ Department of Urology University of Tsukuba Hospital Tsukuba Ibaraki Japan

**Keywords:** imaging, inferior, retroperitoneal neoplasm, three‐dimensional, vena cava

## Abstract

**Introduction:**

A double inferior vena cava is a rare anomaly with an incidence ranging from 0.3% to 3.0%. In patients with a double inferior vena cava, it is important to understand the precise anatomy and possible irregular lymph node flow when performing surgery for malignancies.

**Case presentation:**

A 60‐year‐old man with a non‐seminoma was referred to our hospital after left high orchiectomy. Computed tomography revealed a double inferior vena cava and swollen masses in the para‐aortic region. After four cycles of chemotherapy with etoposide and cisplatin, retroperitoneal lymph node dissection was safely performed with a modified template extended to the right side of the paracaval region by referring to three‐dimensional images created by SYNAPSE VINCENT® software.

**Conclusion:**

Preoperative three‐dimensional images were useful to understand this patient’s unusual and complicated anatomical positions.

**Abbreviations & Acronyms**
3D: three‐dimensional
CT: computed tomography
IVC: inferior vena cava
RPLND: retroperitoneal lymph node dissection

**Keynote message**

Preoperative 3D images were important to understand in a patient with double IVC. Careful inspection and discussion are necessary for unusual and complicated anatomical positions before an intensive operation.

## Introduction

A double IVC and left IVC are congenital anomalies caused by abnormal fusion or retraction during the process of vena cava development, and their incidence ranges from 0.3 to 3.0%.^1^ They are generally asymptomatic and usually incidentally found during imaging examinations. In patients with such IVC anomalies, treatment strategies for retroperitoneal surgery must be based on a precise anatomical understanding. We herein report a case of metastatic left testicular cancer in a patient with a double IVC and describe the usefulness of 3D images for RPLND.

## Case presentation

A 60‐year‐old man visited another hospital because of left scrotal enlargement. The testis was swollen and stiff on palpation, and ultrasound demonstrated a mosaic echoic mass. Blood examination showed an elevated human chorionic gonadotropin concentration of 6418 mIU/mL (reference range, <0.5 mIU/mL), elevated alpha‐fetoprotein concentration of 3534.7 ng/mL (reference range, 7–10 ng/mL), and normal lactate dehydrogenase concentration of 189 U/L (reference range, 106–211 U/L). Contrast‐enhanced CT showed normally sized lymph nodes and a double IVC. Left high orchiectomy was performed, and the pathological diagnosis was non‐seminoma pT2 (60% embryonal carcinoma, 20% yolk sac tumor, and 20% immature teratoma) with vascular invasion. The patient’s tumor markers did not normalize postoperatively, at which point he was referred to our hospital. Blood examination in our hospital showed a human chorionic gonadotropin concentration of 46.4 mIU/mL, alpha‐fetoprotein concentration of 59.9 ng/mL, and lactate dehydrogenase concentration of 141 U/L.

**Fig. 1:**
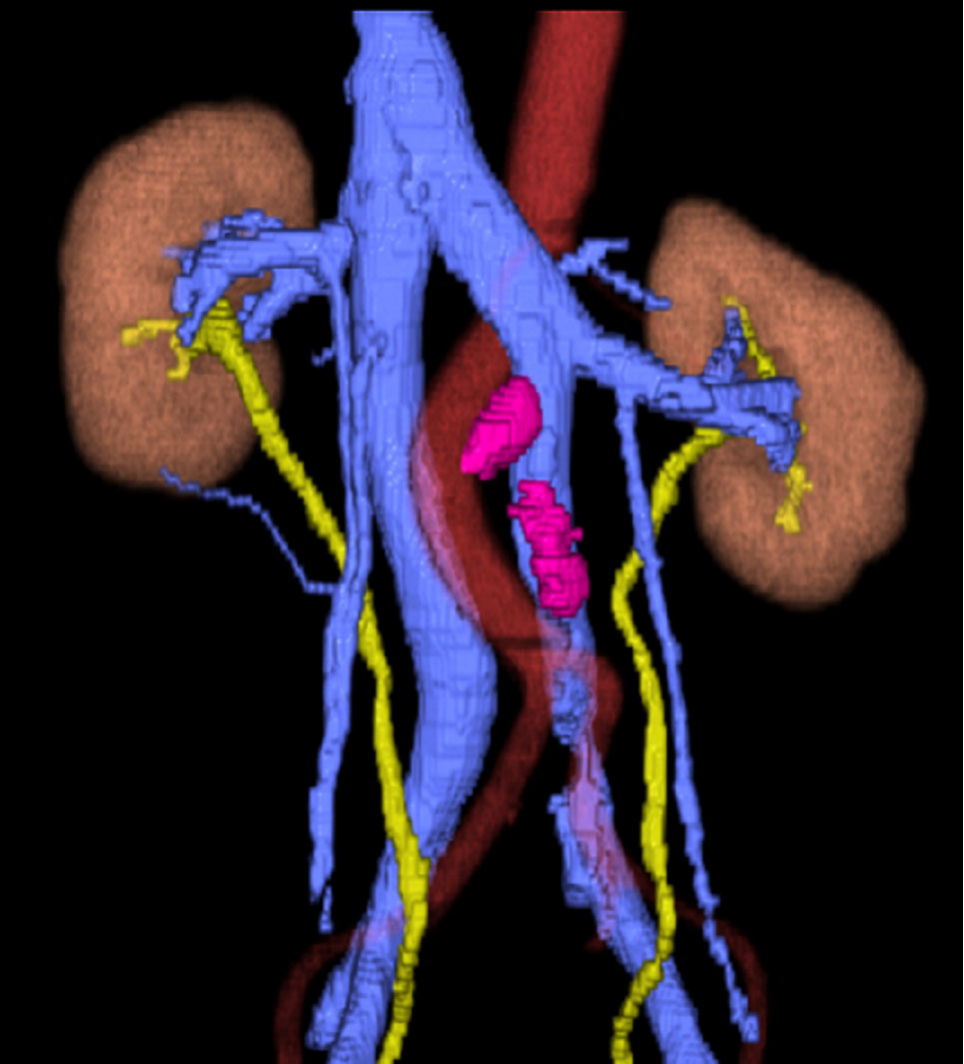
3D images of vessels, ureters, and tumor created by SYNAPSE VINCENT® software.

**Fig. 2:**
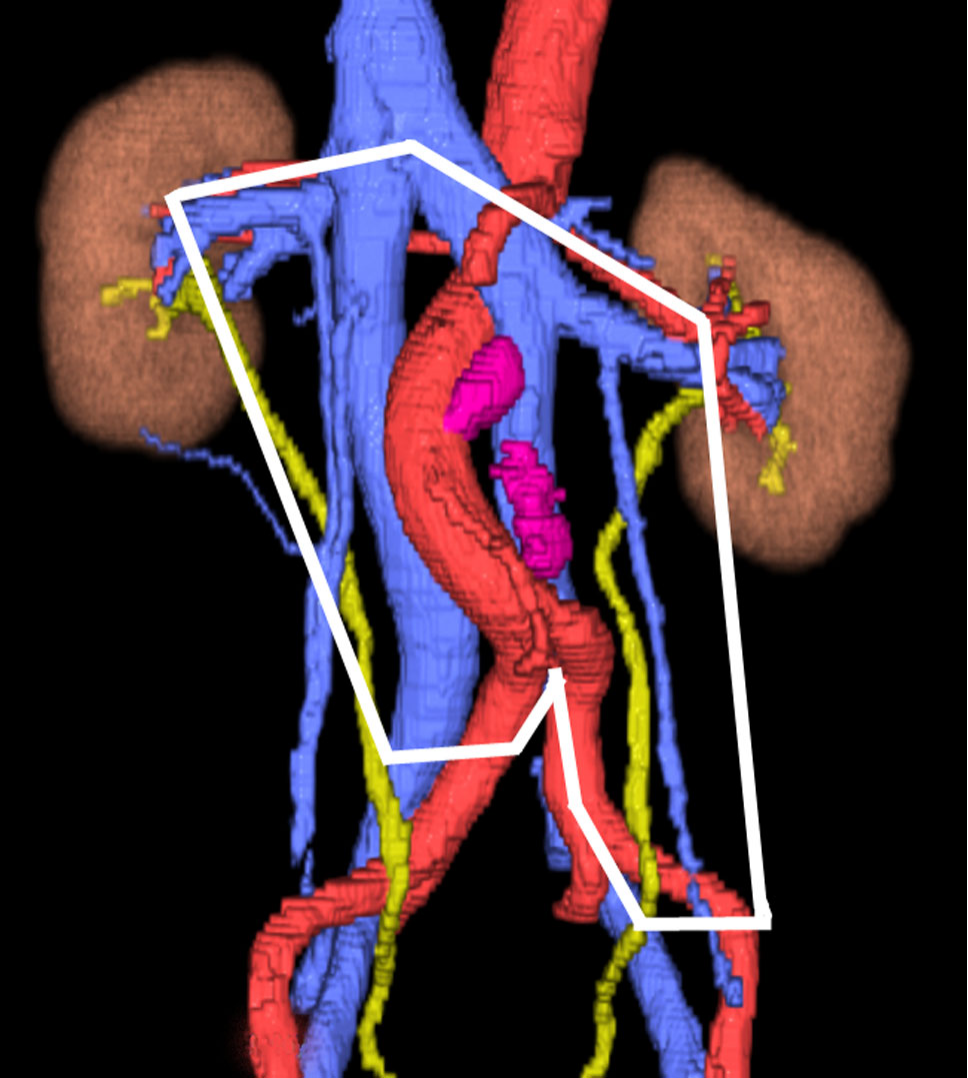
Dissection range of retroperitoneal lymph nodes in this case.

**Fig. 3:**
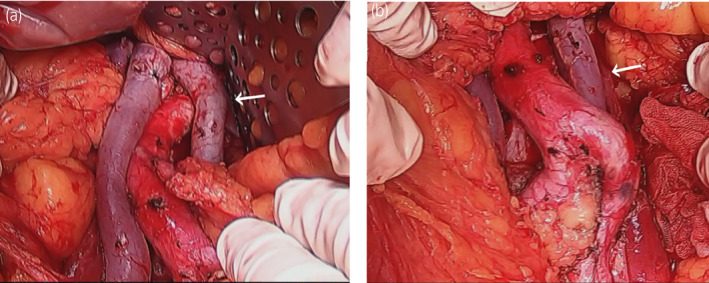
Intraoperative images of double IVC. (a) Upper field from renal hilum to resected inferior mesenteric artery. (b) Lower field from resected inferior mesenteric artery to aortic bifurcation. The arrow indicates the left side of the double IVC.

CT performed 1.5 months after the initial CT examination revealed swollen lymph nodes in the para‐aortic region (under the left renal hilum, at the bifurcation of the inferior mesenteric artery). The clinical diagnosis was left testicular cancer T2N1M0S2, Stage IIA according to the Japanese Urological Association; the prognosis was considered to be good according to the International Germ Cell Consensus Classification. After four courses of chemotherapy with etoposide and cisplatin, the tumor marker levels decreased to the reference range and the swollen lymph nodes decreased to a maximum diameter of 1.0 cm. Therefore, we planned to perform RPLND. Because of the double IVC, we used the preoperative CT images to create 3D images using SYNAPSE VINCENT® software (Fujifilm Medical Co., Ltd., Tokyo, Japan) (Fig. 1). The 3D images clearly showed that the left iliac vein formed the left IVC, which ascended along the left side of the abdominal aorta and flowed into the left renal vein. No findings of an interiliac vein were identified. Residual lymph node masses were located under the left renal hilar region and at the bifurcation of the inferior mesenteric artery, and these lymph node masses were in contact with the left IVC. Because anomalous lymphatic drainage associated with the double IVC was highly suspected,^2^ we decided to extend the template of the dissection range to include the right side of the right IVC (Fig. 2). Intraoperative examination revealed that the residual lymph node masses were in contact with the aorta and left IVC, as the 3D images indicated, and that severe adhesion with the surrounding tissues was absent (Fig. 3). The operation time was 5 h 40 min, and the intraoperative blood loss was 1112 mL. The postoperative course was uneventful, and the patient was discharged from our hospital on postoperative day 14. The surgical specimen included a teratoma component, and no recurrence was observed during 17 months of follow‐up.

## Discussion

Anomalies of the IVC are now found more frequently with recent developments of imaging modalities, and testicular cancer is associated with a somewhat higher frequency of IVC anomalies.^3^ It is quite rare for urologists to encounter a double IVC with concurrent metastatic testicular cancer.

IVC development is very complicated, making it prone to various venous anomalies such as double IVC, IVC defect, and left IVC.^1^ A double IVC develops when a connection between the sacrocardinal vein and left subcardinal vein does not disappear, and an interiliac vein is often present.^4^, ^5^

The interiliac vein derives from the left common iliac vein and connects the left and right IVC, and often passes the dorsal side of the abdominal aorta, which can increase the risk of intraoperative bleeding complications.^1^ Therefore, it is important to confirm the presence or absence of an interiliac vein before and during surgery for patients with double IVC. In addition, operators should pay attention to the left gonadal vein. Because the left gonadal vein normally develops from the left subcardinal vein, the left gonadal vein runs along the lateral side of the left IVC. This increases the risk of misidentification between the left IVC and the left gonadal vein.^1^, ^5^ To avoid this misidentification, operators are recommended to follow the vein distally to confirm whether it derives from the gonad or the iliac vein.

Although little is known of the lymphatic flow of the double IVC, the lymphatic development is considered to have a similar asymmetrical regression pattern as the venous system and aberrant or interiliac lymphatic drainage might exist.^6^ Therefore, it is important to consider a wider dissection range including the contralateral side as a full template in metastatic testicular cancer patients with double IVC.

A preoperative understanding of anatomy is essential for a safe operation, especially in such cases with unusual and complicated anatomy. For this reason, we performed a 3D analysis using SYNAPSE VINCENT® software based on the patient’s CT images. There are only a few reports showing preoperative 3D images in RPLND,^7^ while the utility of 3D images was evaluated in other surgeries.^8^, ^9^, ^10^, ^11^ This technique enabled us to visualize the positional relationship of the abdominal aorta and left IVC to the lymph node masses to gain a precise understanding of this uncommon anatomy by freely rotating the 3D images, and to define the dissection range according to the stereoscopic blood vessel route consistent with the surgical view. As a result, the surgery was performed safely.

## Conflict of interest

The authors declare no conflict of interest.
